# Mitochondria Have Made a Long Evolutionary Path from Ancient Bacteria Immigrants within Eukaryotic Cells to Essential Cellular Hosts and Key Players in Human Health and Disease

**DOI:** 10.3390/cimb45050283

**Published:** 2023-05-19

**Authors:** Anna Atlante, Daniela Valenti

**Affiliations:** Institute of Biomembranes, Bioenergetics and Molecular Biotechnologies (IBIOM), National Research Council (CNR), Via G. Amendola 122/O, 70126 Bari, Italy

**Keywords:** mitochondria, ancient bacteria, mitochondrial energy metabolism, mitochondrial dysfunction, cardiolipin, mitochondria-related disorders, cell death

## Abstract

Mitochondria have made a long evolutionary path from ancient bacteria immigrants within the eukaryotic cell to become key players for the cell, assuming crucial multitasking skills critical for human health and disease. Traditionally identified as the powerhouses of eukaryotic cells due to their central role in energy metabolism, these chemiosmotic machines that synthesize ATP are known as the only maternally inherited organelles with their own genome, where mutations can cause diseases, opening up the field of mitochondrial medicine. More recently, the omics era has highlighted mitochondria as biosynthetic and signaling organelles influencing the behaviors of cells and organisms, making mitochondria the most studied organelles in the biomedical sciences. In this review, we will especially focus on certain ‘novelties’ in mitochondrial biology “left in the shadows” because, although they have been discovered for some time, they are still not taken with due consideration. We will focus on certain particularities of these organelles, for example, those relating to their metabolism and energy efficiency. In particular, some of their functions that reflect the type of cell in which they reside will be critically discussed, for example, the role of some carriers that are strictly functional to the typical metabolism of the cell or to the tissue specialization. Furthermore, some diseases in whose pathogenesis, surprisingly, mitochondria are involved will be mentioned.

## 1. Mitochondria: Ancient Immigrants within Eukaryotic Cell

Mitochondria work hard. They produce nearly fifty kilograms of adenosine triphosphate (ATP) every day. According to the English biochemist and writer Nick Lane, “Gram per gram, even when sitting comfortably, you are converting 10,000 times more energy than the sun every second” [1].

**Figure 1:**
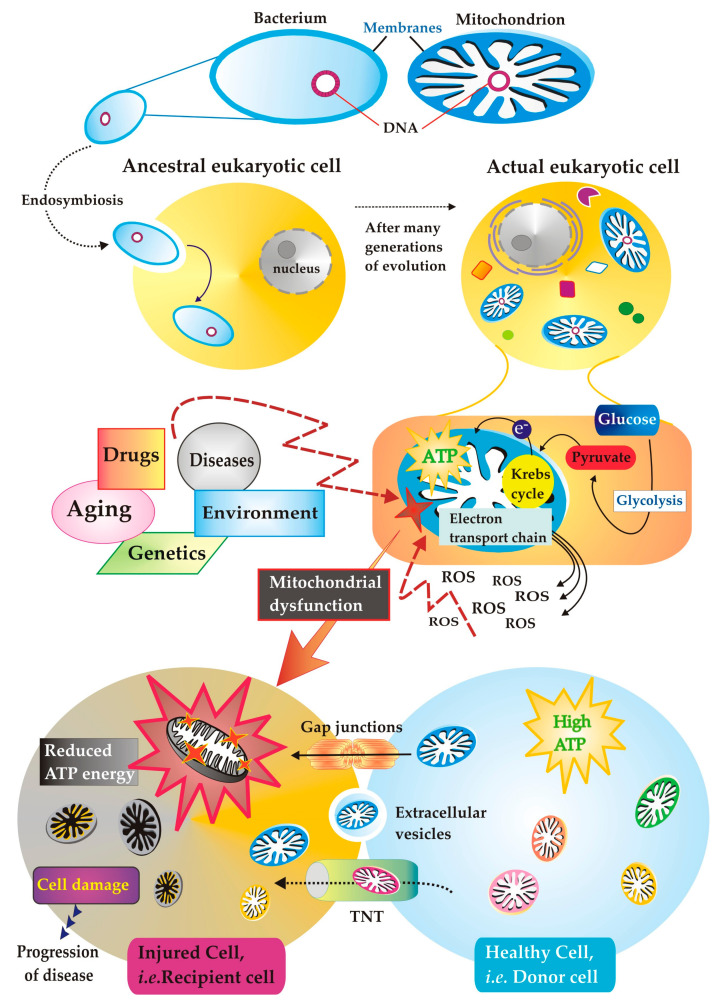
In the upper part of the figure, a descriptive path taken by the mitochondrion is reported: as an ancient bacterium enters the ancestral eukaryotic cell specializing in an organelle dedicated to energy production but also a target of ROS—which it produces itself—as well as of effectors, such as drugs, diseases, the environment, aging, or genetics, that cause it to malfunction. In the lower part of the figure, the transfer of mitochondria between cells is described: the healthy cell (donor cell) donates healthy mitochondria to the injured cell (recipient cell) containing dysfunctional mitochondria. Abbreviations: ROS, reactive oxygen species; TNT, tunneling nanotube.

How these precious organelles form inside cells is a mystery. There is something unique and special about mitochondria that clearly distinguishes them from all other cellular organelles, i.e., the fact that they contain their own DNA, which is very similar to bacterial DNA. In fact, the most accredited hypothesis traces the origin of the mitochondrion to a sort of infection of the ancient eukaryotic cell, which millions of years ago managed not only to survive but even to swallow, without destroying it, an aerobic bacterium that knew how to deal with oxygen, i.e., being able to use oxygen to produce energy [2], establishing a symbiotic relationship of mutual benefit (Figure 1).

The idea that mitochondria originated from free-living bacteria that were incorporated within an archaic eukaryotic cell via endocytosis [3] is supported by phylogenetic analysis of conserved ribosomal RNA (rRNA) [4,5]. Thus, over time, against all possible odds, the two cells, so different, have learned to coexist, establishing a symbiotic relationship: the intruders have given the eukaryotic cells something special, i.e., an extra set of DNA, mitochondrial DNA (mtDNA), which cells need to get a constant supply of energy. We now know that if you lose that DNA, catastrophic consequences ensue (see below). Therefore, as ancient immigrants, the mitochondria have taken their revenge, representing the very essence of cell life: in fact, they not only produce energy, but their role is decisive for various cellular processes such as homeostasis, responses to stress, cell survival, and much more. In particular, the ability to use oxygen, characteristic of that engulfed bacterium, has been preserved and refined in the organelles: mitochondria are the site of aerobic respiration and ATP production, i.e., the energy that allows cells to do what they do when they are alive.

Oxidative phosphorylation (OXPHOS), the process by which the mitochondria produce ATP, is also the main process generating reactive oxygen species (ROS) (Figure 1), which play important roles as cellular signal mediators but can nevertheless be extremely dangerous for the cell when their production becomes uncontrolled; the very integrity of mtDNA can be endangered precisely by the action of ROS. About 1100 mitochondrial proteins are encoded by nDNA [6], translated in the cytosol as precursors of mature proteins [7], and recognized, thanks to specific targeting signals, by receptors present on the mitochondrial surface. The mitochondrial proteins encoded by mtDNA, although constitute only a small fraction of the subunits of all respiratory enzymes [8], are essential for the proper functioning of OXPHOS.

Quite frequently, the mitochondria rise to the headlines due to some discovery concerning them, in virtue of the multifaceted activities they perform, and there are not a few scientists who find mitochondria intriguing due to their peculiar nature and fascinating biology. In the marvelous animated world of Albert Barillé, educational cartoonist par excellence, the mitochondria assumed, through a fantastic representation, the contours of imposing industrial establishments. In the small space of the organelle, there are reagents, metabolic intermediates, and catalysts (enzymes); balances are calculated regarding the exchanges of matter and energy (its role is also essential in the production of heat); and there is also fear of some accident that could cost one’s life. There are also transport systems, analogous to tank trucks and freight trains, as Barillé depicts them, which we will discuss later.

The first to notice them in 1856 was the Swiss physiologist and anatomist Albert von Kolliker [9], who observed granular structures in the cells of muscle tissue, but it was Carl Benda in 1898 [10] who coined the term mitochondrion, derived from the fusion of “mitos”, thread, and “chondros”, grain, of which they recall its shape. About a century after their discovery, the Nobel prize for medicine goes to Sir Hans Adolf Krebs for the discovery of a cycle of reactions, i.e., precisely the Krebs cycle, which takes place in the mitochondria of eukaryotic cells and identifies the role of the citric acid cycle in the intermediate metabolism [11].

How ATP molecules, the body’s main fuel, were formed in the cell in the presence of oxygen but not in its absence, remained to be “deciphered”. Scientists were familiar with electron transporters (NADH and FADH_2_) and knew that these coenzymes transfer high-energy electrons to a series of molecules present in the inner membrane of the mitochondria, which, as linked rings, constituted an electron transport chain. In practice, they understood that the respiratory chain (RC) consumes the oxygen present in the cell, generating ATP [12,13], but they had no idea of the mechanism by which ATP was formed. Thus, scientists started looking for reaction intermediates and enzymes that would catalyze the reactions until, in 1961, the English biochemist Peter Mitchell proposed a mechanism that coupled the transfer of electrons to the synthesis of ATP through a process that was called chemiosmosis [14].

Here, however, we have a problem: defects in the transfer of electrons across the mitochondrial membrane can cause the accumulation of electrons on the RC complexes, which leads to the formation of substances whose names alone inspire fear and trembling: ROS [15,16,17,18]. ROS are extremely reactive, capable of stripping electrons from the first molecules that come within range, triggering chain reactions that can propagate oxidative damage to molecules such as lipids, proteins, and even DNA. The rapid propagating succession can be stopped only when two radicals react with each other to form stable compounds or by the action of antioxidants, compounds capable of giving up electrons and quenching the insatiable thirst for ROS, thus interrupting the inauspicious chain. Between 0.2 and 2% of the oxygen absorbed by cells is converted into ROS [19].

The accumulation of electrons increases the potential for electrons to bind to free oxygen species, thereby contributing to a wide variety of pathological conditions, including degenerative diseases, cancer, and aging [20]. Brand hypothesized that increased mitochondrial uncoupling, which helps dissipate the electrochemical proton gradient, reduces ROS production, thereby protecting mitochondria and other cellular proteins and lipids from superoxide radical damage [21]. Supporting this hypothesis, Speakman et al. demonstrated that increased mitochondrial uncoupling was related to increased lifespan in rodents [22]. Therefore, conditions that reduce the proton gradient (uncoupling, caloric restriction) are thought to be protective against conditions or diseases related to oxidative stress (aging, diabetes mellitus, some forms of cancer).

In his pioneering work, spanning nearly two decades, Mitchell theorized an anisotropic arrangement of components involved in ATP synthesis within the inner mitochondrial membrane impermeable to protons so that the RC complexes could transfer electrons and expel protons across the membrane. Mitchell’s chemiosmotic theory, which earned him the Nobel Prize in 1978, shaped the basis for the complete understanding of the processes of OXPHOS. This exemplary example of basic research should lead us to reflect on the importance of studies having as their objective knowledge of the mechanisms that regulate biological processes, which constitutes the fundamental and indispensable prerequisite without which there would be no foundations for future applications.

A representative and salient example is the clinical treatment of a disease. It makes us understand that basic research cannot be disregarded; otherwise, one would make big mistakes in the ‘packaging’ of the therapy to be used as a cure for a disease, or at least in treatments that could contain its symptoms. Therefore, as far as basic research, which seems to be so far from the history of the patient, could become critical when the possibility of using the scientific discoveries of basic research in clinical applications arises.

Therefore, in the course of evolution, thanks to the symbiotic relationship created, cells relied on mitochondria for energy production and mitochondria on cells for their own subsistence. In fact, the mitochondria communicate with the cytoplasm to implement in the cell an intense traffic of metabolites, proteins, and cofactors towards and from these organelles, so much so that variations in mitochondrial functionality are reflected in the biochemistry of the whole cell.

In short, the mitochondria perform key multitasking roles, so much so that the organs that require more energy to function, such as the brain, heart, and muscles, are the ones richest in mitochondria. For example, the cells of the heart, an energy-intensive organ, contain up to 40% mitochondria [23]. Each of us has quadrillions of them, and each of them biosynthesizes ATP. It follows that if the transmission of ATP to the remaining part of the cell is interrupted or compromised, the road to physical and cognitive decline could be short. The good news is that this process could be reversible, so restoring the communication between mitochondria and cells can, if performed in time, not only interrupt but also reverse the effects of the decay [24,25]. Energies, metabolism, and reasoning skills will be boosted, while diseases such as Alzheimer’s (AD), heart attack, or even cancer could be kept at bay.

However, although “the powerhouse of the cell” is surely the first memorable phrase in biology concerning mitochondria, an explosion of new information about mitochondria reveals that their importance goes far beyond their ancient function as the “powerhouse of the cell” [26,27]. Suffice it to say, in fact, that they have a fundamental responsibility in preserving our vital functions and maintaining our state of good health; any dysfunction involving them could lead to a pathological status. This is why mitochondrial research continues to be feverish around the world: a growing number of studies place mitochondria at the center of cellular life and mitochondrial dysfunction at the heart of disease.

We are not only talking about specific mitochondrial diseases, i.e., those of a hereditary nature, which we will discuss briefly below, but also about the fact that various factors of mitochondrial origin are the basis of many disorders. In particular, dysfunctions of mitochondrial structure and function are associated with autism spectrum disorders and metabolic, cardiovascular, renal, and neurological diseases (e.g., Alzheimer’s and Parkinson’s) [28,29,30]. Again, no one would have suspected, for example, a specific mitochondrial involvement in the progression of cystic fibrosis [31]. However, it does not end there, because the role of mitochondria in immunity also makes them important for counteracting autoimmune diseases [32,33]. Furthermore, the accumulation of mitochondrial defects has been implicated as a pivotal mechanism of aging and age-related diseases [34], and, not least, mitochondria have emerged as central players in the regulation of programmed apoptotic cell death [35], whose inappropriate activation leads to tissue dysfunction and damage as well as, conversely, the lack of activation leads to cancer. Thus, while they provide energy for the cell by serving as a hub for biosynthetic processes, they also contain a self-destructive arsenal of apoptotogenic factors that can be released to promote apoptotic signaling.

Reflecting on the fact that the biology of the mitochondria is assuming ever-increasing relevance in modern medicine [36,37], as many processes underlying disease actually match what happens in the mitochondria, it is not surprising that since mitochondria have enormous potential to influence health, optimizing their metabolism could indeed be at the heart of effective therapeutic treatment. This, in fact, is a research field undergoing great development. The discovery that some natural molecules are capable of correcting mitochondrial dysfunction, or at least reducing it, is promoting research for the identification of new molecules that can be used as drugs capable of correcting mitochondrial dysfunction, if not actually preventing it [38,39].

From what has been said so far, that ancient bacterium, a guest of the eukaryotic cell, undoubtedly made itself useful at home. In this regard, although it is important to keep in mind that mitochondria have various functions, both vital and providentially fatal (see apoptosis), it is nevertheless true that the mitochondrial “raison d’etre” is the production of energy as ATP in both cases.

Furthermore, mindful of their bacterial origin, the mitochondria—differently from how they are illustrated almost everywhere, in scientific books and magazines, as rounded, often ovoid little creatures, like beans—not only move but touch, unite, and separate continuously, like bees in a hive. They move freely in the cytoplasm on intracellular tracks, which are the microtubules, pulled by locomotives, which are motor proteins, often GTPases, and tend to gather where there is a greater energy demand [40,41,42]. The importance of this motility becomes clear, at least to neurologists, when one considers a motor neuron in the spinal cord that sends its axon to a distal muscle: that motor neuron’s mitochondria have a long way to go from the soma to the neuromuscular junction [43,44]. Regarding the fission and fusion processes, the mitochondria stretch and contract, fuse with each other, and divide again, so much so that they are defined as social organelles, being able to communicate and synchronize their respective activities and react both to their surroundings and to each other [45]. This is not surprising when one considers that bacteria are among the simplest living organisms, yet they exhibit remarkable community behaviors. The purpose of these meetings is certainly collaborative; in fact, mitochondria that lack a genome or whose genetic material is dysfunctional can be “saved” by resorting to fusion with healthy organelles, just as it is possible that fusion helps distribute proteins more equally among the various organelles. Recent discoveries prove that mitochondria communicate and collaborate with each other, not only within the same cell but also between different cells, thanks to their ability to come to the rescue of dysfunctional mitochondria that are inside other cells through their release, thus influencing the health of the whole organism (see below). In 2016, Khacho and colleagues reported the first evidence that mitochondrial shape change is a key regulator of neural stem cell fate [46]. By knocking out genes encoding proteins key to fusion and fission machinery in mice, the authors found that a lack of fusion proteins reduces the ability of neural stem cells to reconstitute themselves and encourages the cells to become neurons. Exactly how mitochondrial shape change may control cell fate decisions is an open question.

In this critical review, we will describe just some of the research areas on mitochondria that scientists have explored. Bearing in mind that the ancient bacteria are not only still there but have even become precious guests, we will focus on certain particularities of the organelle, for example those relating to its metabolism rather than its energy efficiency, which are of absolutely vital importance and have not been covered in textbooks to date. Above all, we will focus on those mitochondrial ‘novelties’ that, although they have been discovered for some time, are still not kept in mind today in the progress of research. We would dare to define them as ‘neglected innovations. Moreover, we will talk about their specific function, which reflects the type of cell in which they reside; for example, the role of carriers is strictly functional to the typical metabolism of the cell or, better yet, to the specialization of the tissue. The ability of mitochondria to expel proteins into the cytosolic environment will also be addressed, as will their capability to decide themselves to abandon the cell that up to that moment had hosted them or even to fall asleep, that is, to no longer be sensitive to the surrounding environment. Furthermore, we will also review certain diseases in which, surprisingly, mitochondrial dysfunction has been found to be critically involved.

## 2. Peculiarities of the Structure and Function of Mitochondria: What You Need to Know, but No One Knows, or It Is Known, but It Is Ignored

### 2.1. What Is Important to Know about Mitochondrial Membrane Structure: The Peculiar Presence of Cardiolipin

We report below a brief description of the mitochondrion to give the reader, who knows little about it, the opportunity to get an idea of mitochondrial biology. Mitochondria are delimited by two membranes, whose architecture reflects their functional specialization: an outer mitochondrial membrane (OMM) and an inner one (inner mitochondrial membrane, IMM). OMMs are formed by phospholipids and proteins that have a transport function and allow the passage of material between the cytoplasm of the cell and the mitochondria. IMM is also made up of proteins and phospholipids, with a high presence of cardiolipin (CL), a peculiar and essential component of this membrane, where it plays a fundamental role in a large number of processes [47,48,49] (see below). CL accumulates mainly in the folds—called cristae—of the IMM, significantly increasing its surface area; moreover, this high presence of CL in the IMM raises the degree of folding of the cristae themselves. It is also interesting to know that the mitochondria richest in CL are also the most efficient at producing energy. Furthermore, CL is involved in mitochondrial cristae morphology and stability [50,51,52], mitochondrial quality control and dynamics through fission and fusion [53,54], mitochondrial biogenesis and protein import [55,56], and mitophagy [46,57,58], as well as in apoptosis, serving as a binding platform for engaging apoptotic factors [46,57,59]. Abnormalities in CL content, composition, and level of oxidation negatively impact mitochondrial function and dynamics, with important implications in a variety of pathophysiological situations and diseases (for refs see [46,60,61]).

The proteins, of which the IMM is very rich, have a transport function; some, assembled in protein complexes, are used for the transfer of electrons, are involved in the production of energy, and are located in the internal introflexion of the cristae [62,63,64].

The space between the two membranes, i.e., intermembrane space (IMS), has an important role in the ATP synthesis processes, while the internal compartment of the mitochondria, called the matrix, contains a large number of enzymes involved in various metabolic pathways: urea cycle, production of the heme group, Krebs cycle, and β-oxidation of fatty acids. It is here that the “treasure” of mitochondria is contained, i.e., their own genetic material, mitochondrial DNA, which makes them unique among all cellular organelles.

The main role of the mitochondria is to convert the products of carbohydrate, protein, and fat metabolism into CO_2_ and water using key enzymes of the RC: NADH dehydrogenase (complex I), succinate dehydrogenase (complex II), cytochrome *bc*1 (complex III), and cytochrome *c* oxidase (complex IV). In these respiratory complexes, electrons from the oxidation of carbohydrates and fats, collected by specialized molecules, the coenzymes NADH and FADH_2_, are passed from one complex —which oxidizes —to the next —which reduces —to expel protons (H^+^) in the IMS, and finally transferred to oxygen with the formation of water. Only a specific localization of the RC proteins in the lipid bilayer allows the vectorial translocation of the proton during the flow of the reducing equivalents towards oxygen [13,65,66]. Since IMM is impermeable to protons, an electrochemical proton gradient is formed across the membrane [13], which can be used to import proteins and Ca^2+^ into the mitochondria, generate heat, and synthesize ATP. The cascade of protons, flowing back from the IMS towards the matrix, supplies energy to a protein—ATP synthase FoF1 (respiratory complex V) [67]. The FoF1 complex acts as a turbine capable of using the electrochemical gradient, i.e., the proton gradient between the IMS and the mitochondrial matrix, to produce almost all the ATP needed by the cell. It is this coupling of substrate oxidation with ATP formation in the mitochondria that is termed OXPHOS [13,68,69] and is critical to the health of a variety of tissues and organs. Strictly speaking, it is more accurate to define protein complexes as ‘lipoprotein complexes’ because lipids, in particular CL, are essential for their structural and functional organization and also for keeping the protein molecules properly oriented in the bilayer. Regarding the CL, due to its high content of unsaturated acyl chains and its location in the IMM close to RC complexes, which are major ROS production sites, CL molecules are readily susceptible to ROS attack, causing peroxidation, an event that can influence many CL-dependent reactions and processes [46]. Specific binding sites for CL have been detected in complexes I, III, and IV [46,69]. Furthermore, an active role for CL in proton translocation at the level of complex III and IV has been proposed [70].

Matrix ATP is then exchanged for cytosolic ADP via the IMM adenine nucleotide translocator (ANT) [71,72]. It seems that ANT, a basic protein and therefore rich in positive charges, also balances the excess of positive charges by interacting with CL, which is rich in negative charges [72]. Klingenberg demonstrated that ANT activity is optimal only in the presence of tetralinoleoyl-CL, while other CL species and other phospholipids are ineffective in stabilizing ANT activity [73]. Furthermore, crystallographic studies suggest that there are two or three CL molecules tightly bound to the monomer of this protein [74]; these CL molecules favor the stabilization of the dimeric structure of ANT, which is the active form of this protein [73].

A singular event that occurs during apoptosis is the redistribution of CL from the IMM to the OMM [75,76], so much so that CL can be considered a mitochondrial stress signaling factor with a specific role in apoptotic and mitophagic pathways. Externalized CL interacts with cytochrome *c* (cyt *c*), forming a cyt *c*/CL complex [76,77], which exhibits peroxidase activity [78] capable of targeting the unsaturated acyl chains of CL, resulting in an increase of oxidized CL [79] and, consequently, the detachment of cyt *c*, which shows a lower affinity towards oxidized CL [80], and its release from the mitochondria to the cytosol. The same cyt *c*, whose action in the RC has been well known for several decades, i.e., a shuttle that transfers electrons from complex III to complex IV [76,81], has a novel function that is exerted outside the mitochondria and is not related to its own redox function. In the early stages of cellular apoptosis, cyt *c*, released into the cytosol, binds to apoptotic protease activating factor-1 (Apaf-1) and forms the apoptosome, a complex that activates a caspase cascade leading to cell death [76,82].

Despite the large body of literature dealing with the release of cyt *c* from mitochondria in cells undergoing apoptosis [83,84,85] and its role in caspase activation [85,86,87], Atlante et al. [88,89] demonstrated that cyt *c* is released from the coupled and intact mitochondria of cerebellar granule cells (CGC) as an intact and functionally active protein. In this active state, cyt *c* acts as a scavenger of ROS and as a respiratory substrate. Released cyt *c* is capable of causing proton expulsion and therefore the generation of electrochemical membrane potential, driving the synthesis of ATP and its export in the extra-mitochondrial phase. Thus, the released cyt *c* can contribute to the supply of ATP necessary for programmed cell death [89]. Therefore, it is able to accept electrons from ROS, deliver them to COX, and drive ATP synthesis [89]. However, direct oxidation of cyt *c* by COX at mitochondrial contact sites [90] seems unlikely given the structure of the oxidase and its apparent inability to span the two mitochondrial membranes. This cross-talk between life and death is quite complex and sometimes contradictory, but certainly critical for the overall fate of the cell. Furthermore, cross-talk is a key factor in the outcome of several death-related pathologies, such as cancer and neurodegenerative processes. That programmed cell death (i.e., apoptosis) is a mechanism that is generally lost by tumor tissues was observed by Albano et al. [91] in advanced melanoma cells, in which the expression of the Bcl-2 protein (present in at least 80% of the cases examined) in abundance blocks apoptosis. This block occurs because Bcl-2 prevents the release of cyt *c* from the mitochondria in response to the damage induced by chemotherapy, supporting both the hypothesis that cyt *c* is the true director of cellular apoptosis and that the blockade of the expulsion of cyt *c* by Bcl-2 is the leading cause of chemotherapy resistance in melanoma disease [91].

### 2.2. Specificity of Mitochondrial Translocators: Their Function Is Dependent on the Metabolism of the Tissue in Which They Operate

In their complexity, the four compartments, although having different biochemical characteristics, have interdependent and coordinated functions useful for sustaining the cellular energy metabolism. The outer membrane, characterized by a high lipid/protein ratio, is essentially smooth and somewhat elastic and contains a channel for anions, the voltage-dependent anion channel (VDAC), also called porin, whose activity is dependent on the electric potential and regulated under physiological conditions by a soluble protein located in the IMS [92,93]. Precisely because it is delimited by a membrane, the ability of the ancient bacterium to cooperate in the family organization of the cell is strictly dependent on the possibility of selectively receiving some metabolites from the cytoplasm and exporting others to the outside. In fact, the different localization of certain substrates and of the enzymes responsible for their metabolism requires that the cytoplasm and the internal compartments of the mitochondria be put in communication through carrier-mediated transport processes. Brian Chappell was the first to have identified in the sixties of the twentieth century the existence in the mitochondria of protein-mediated transport processes [94] that allow the integration and completion of important metabolic processes taking place between the mitochondria and the cytosol, thus participating in the trafficking of metabolites, proteins, and cofactors to and from these organelles. Over the past forty years, new carriers and/or new transport processes have been discovered, all belonging to the same ‘family’ by virtue of the common characteristics of their protein molecules [95,96]. In this regard, in all our long years of research, our goal has always been to investigate the existence of transport proteins in intact organelles so as to better understand their physiological role. Not only that, over the years, this goal of ours has also been pursued in more complex experimental systems, such as the homogenate of cultured cells, which, compared to a sample of isolated mitochondria, contains very small amounts of mitochondria. The miniaturization of techniques already used previously to study the transport of metabolites in the mitochondria in almost physiological conditions, i.e., intact respiration and phosphorylating mitochondria, has represented a difficult obstacle to overcome, but certainly with a winning outcome (for refs, see [97,98,99]).

Using isolated and coupled mitochondria, the experimental strategy that leads to the identification of a translocator can have various starting points. In some cases, having localized inside the mitochondria one or more specific enzymes catalyzing reactions in which the substrate is a metabolite produced in the cytosol, it occurs to characterize the transport process across mitochondria, allowing the metabolite to reach its/their intra-mitochondrial enzymes. Sometimes, the experimental evidence that a given metabolite enters the mitochondria requires the study of its intra-mitochondrial metabolism. This type of experimental investigation could lead to the discovery of new enzymatic reactions and metabolic steps. This is the case of the reaction catalyzed by L-lactate oxidase, a flavin enzyme identified and localized in the IMS of rat liver mitochondria (RLM) [100]. de Bari et al. arrived at the identification and characterization of this mitochondrial enzyme by monitoring the production of hydrogen peroxide (H_2_O_2_) with 1:1 stoichiometry with pyruvate as a result of L-LACT addition to isolated RLM. The putative L-lactate oxidase exhibited a hyperbolic dependence on L-LACT concentration, which was shown to be competitively inhibited by NAD(+) and different from L-lactate dehydrogenase isoenzymes, as shown by their different pH profiles [100].

The challenge of measuring transport was essentially represented by the method to be used to follow the entry of a metabolite into the mitochondria or its efflux from them.

A meticulous method for studying the translocation of a metabolite is the one defined as “indirect”: it allows to follow the transport of a metabolite into mitochondria through a reaction associated with the appearance of the same inside mitochondria or revealing its efflux in the extra-mitochondrial phase. The measurements are performed by spectroscopically monitoring the variations in the redox state of the intra- or extra-mitochondrial pyridine coenzymes involved in reactions catalyzed by specific dehydrogenases by following in continuous succession the changes in absorbance or fluorescence that measure the carrier-mediated metabolic trafficking of uptake or efflux across mitochondrial membranes (for refs, see [95]). Just a small digression: the difference between direct methods, usually isotopic, and indirect ones is substantial because, in the first case, all mitochondrial activity is blocked in order to prevent the metabolism of the substrate entering the mitochondria; in the second case, the metabolism is largely possible, and consequently transport processes can be observed caused by the formation of product(s) deriving from the metabolism of the substrate entered into the mitochondria. If you do not block the metabolism through direct methods, you can draw erroneous conclusions. This is the case with fumarate (FUM), which for many years was considered a substrate unable to enter the mitochondria due to the fact that the labeled FUM in the matrix is transformed by fumarase into malate, which flows in exchange with the same FUM. As a result, it would have been impossible to observe the accumulation of radioactivity linked to ^14^C-FUM in the mitochondria (see [94]).

Another technique used by many for the study of membrane transport systems is the reconstitution of artificial membranes by the insertion of purified transport proteins into artificial membrane systems made up of phospholipid bilayers [101,102]. Those most widely used for this purpose are liposomes—spherical vesicles whose diameter varies between 50 and 250 nm depending on the techniques used for the preparation. The transport protein is inserted into the phospholipid bilayer of the liposome, forming the proteoliposome. The reconstituted systems are ‘clean’ systems in the sense that we have everything needed for the study of the protein, i.e., the protein itself inserted into a lipid bilayer that mimics the IMM. Stop. This could help in the molecular elucidation of the process, but the interaction between the transport protein and the liposomes will never be able to mirror the mitochondrial membrane environment, the perfect family habitat that is typical of the mitochondrion/cytosol combination. Even the right orientation of the protein in the lipid bilayer is to be considered essential; just think of the anisotropy of the RC complexes, which is strictly essential (see above). That the transport characteristics in isolated mitochondria and in reconstituted systems differ significantly from each other is a certainty for us because one wonders whether a purified protein actually functions as the true translocator in vivo. In this regard, just to give a case in point, some years ago, in a study on mitochondrial alterations induced by the peptide beta amyloid (Aβ) in AD, we observed the lower activity of Complex IV [16]. Two main observations: (*i*) oxygen consumption supported by the substrate Ascorbate plus TMPD, which allows direct reduction of complex IV via cyt *c*, is impaired in the presence of Aβ; (*ii*) COX activity was found to be inhibited both in CGC homogenate exposed to Aβ and in synaptic-enriched AD brain extracts, allowing us to signal the functional defect at the level of complex IV. However, Aβ does not inhibit the activity of commercially available pure enzymes, likely supporting the hypothesis that a direct interaction may exist with sites of the molecule other than the active one (see [16] and references therein). Therefore, the closer the experimental system is to “nature”, the more reliable the conclusions that research can allow to be drawn. Further, since it has been shown that lipids, CL in particular, play a central role in higher-order association of the constituents of the RC (see above) and that peroxidative damage of mitochondrial membranes affects the activity of Complex IV [16,103], we proposed that ROS-mediated damage of the membrane microenvironment could be responsible for Aβ inhibition of Complex IV [104].

Furthermore, it is also important to take into account the fact that any metabolic pathway—involving enzymes located both in the cytosol and in the mitochondria and transport processes across the mitochondria that mediate the flux of substrates and products of the metabolic pathway—undergoes regulation. It follows that only in vitro experiments carried out with isolated intact and phosphorylating mitochondria allow us to make measurements of transport and related metabolism by identifying the limiting stage and the regulatory role of carrier-mediated transport in the global metabolic process.

At last, but not least, the reason why we have remained faithful, almost stubbornly, to the study of the existence of transport proteins in respiring and coupled organelles is because only this method of study allows us to better understand the physiological role of the translocator in cellular metabolism, a role that textbooks ignore, limiting themselves to an incomplete list of carriers. Moreover, since each cell has its own specific metabolism with a specific role for its mitochondria, it is not correct to transfer the transport characteristics of a substrate that has been studied in mitochondria isolated from an organ or tissue to mitochondria of a different origin [105]. However, the hypothesis that what happens in the mitochondria of a certain origin can also happen in others of a different origin, but with a different role, must be considered acceptable. A case in point is represented by the ornithine (ORN) translocator, whose existence is reported in textbooks only in the liver [106], where the reactions of the urea cycle take place, involving enzymes located in the mitochondria and in the cytosol. Glutamate dehydrogenase, the enzymes of the Krebs cycle, carbamyl-Pi synthetase, and ORN transcarbamylase are located in the mitochondrion, with the remainder of the cycle occurring in the cytosol. This means that for the cycle to proceed, ORN must be transported into the mitochondrion and citrulline exported into the cytosol [106]. Conversely, in the kidney, where the activity of ornithine carbamoyltransferase is very low [107], the metabolism of ORN occurs essentially by means of ornithine aminotransferase, which is highly active [108]. Given the intramitochondrial localization of this enzyme [109] and the existence of a cytosolic pool of ORN, the uptake of ORN in the mitochondria is strictly necessary for its metabolism. The existence of a carrier that mediates the exchange of ornithine with Pi has been demonstrated in kidney mitochondria [110], which could explain the synthesis pathway of arginine [107].

Another concept that has slowly established itself but is totally ignored by biochemistry books is that of mitochondrial micro-compartmentation: microenvironments are created in the mitochondria with a composition of substrates different from that of the surrounding environment. An ever-growing consensus is reaching the concept—first proposed by Murthy and Pande [111]—explaining that transporters mediating the uptake/efflux of substrates and the enzymes metabolizing them are subject to micro-compartmentation, which appears to favor those reactions in which transporters and enzymes are involved. Some enzymes, in fact, are located in the mitochondrial membrane, such as to be close to other proteins, enzymatic and not, and therefore to the products of the reactions, which become substrates for the others in a sort of ‘channeling’: the high concentration in situ favors the rate of the reaction [112]. An example is provided by enzymes whose cofactor is ATP: they can interact with the VDAC on the external surface of the OMM, near the space where the ATP synthesized inside the mitochondria becomes available and exported towards the cytoplasm. The examples of hexokinase and glycerol kinase should be familiar to all: they interact with the porin on the outer surface of the OMM in such a way that these enzymes have preferred access to the ATP generated in the mitochondrion [113]. Another peculiarity is represented by the study on the transport of proline into the mitochondria [114]: the ability of glutamate, which flows out of the mitochondria in exchange for externally added proline via the proline/glutamate antiporter, to re-uptake the mitochondria via its proton-compensated symporter, has been demonstrated. This occurs in virtue of the micro-compartmented higher pool of glutamate measured in the IMS compared to that in the extra-mitochondrial phase [114].

It is clear that these considerations raise doubts about the conclusions drawn from experiments in which the reconstruction of transport processes is attempted using transport proteins and isolated phospholipids in the absence of both metabolic energy and mitochondrial enzymes and with non-physiological concentrations of metabolites.

### 2.3. A Digression on L-Lactate: It Was Considered a Waste Product, but Actually It Is Not

The importance of this paragraph, as well as the main reason that motivated us to raise a discussion on this topic, depends on the consideration that, despite the exponentially growing research on mitochondrial biology, it is truly unacceptable that certain topics are neglected by all biochemistry books, even those published more recently.

Therefore, the intent—perhaps a little presumptuous—is that this digression stimulates the interested reader not to “satisfy” with the notions reported in books and magazines in the sector if they are not updated.

Although we can rightly define the mitochondrion as the theater of action of cellular energy metabolism, in biochemistry textbooks it is still reported that certain metabolic pathways take place, e.g., in the cytosol (see glycolysis or gluconeogenesis), while others, e.g., the β-oxidation of fatty acids, the oxidation of amino acids, and the citric acid cycle, take place within the mitochondrial matrix. In fact, it is incorrect to consider the different compartments as structurally and functionally separate and uncommunicable entities. For example, with regard to glycolysis and most of the reactions of gluconeogenesis, it is reported that they occur in the cytosol of eukaryotic cells, while the oxidation of pyruvate (PYR), the final product of the glycolytic pathway, occurs in the mitochondria [115]. However, if glycolysis converts glucose into PYR and gluconeogenesis, in turn, converts PYR into glucose, the two pathways are obviously subject to coordinated and reciprocal regulation in order not to run into futile cycles. Therefore, the fact that the synthesis of glucose passes through the mitochondrion is not accidental; in fact, the exit of the malate from the mitochondrion—which occurs in the gluconeogenetic process—and its oxidation to oxalacetate via the cytosolic isoenzyme malate dehydrogenase reduce the coenzyme NAD^+^ to NADH, which is strictly required to continue the synthesis of glucose. This, in a broad sense, means that there is always the ‘hand’ of some displacement of metabolites—coming from/or directed to the mitochondria—and that it is essential to know how the metabolism between cytosolic and mitochondrial enzymes is integrated. Complex biochemical pathways are tightly regulated, often closely interdependent, and essential for cellular function. What is generally missing is a discussion on the importance of the combined action of two or more enzymes, mitochondrial and cytosolic, and/or translocators to cooperate in certain metabolic processes [115]. Since mitochondria are the mainstay of this interdependence, we understand why mitochondrial diseases have a significant impact on cellular metabolism. Thus, mitochondria are responsible for various pathological conditions, ranging from less severe cases, i.e., those affecting a single organ, to debilitating (and sometimes fatal) pathologies (see [116,117,118]).

The poor updating of textbooks on which students’ study—biologists and doctors of the coming generation—could be responsible for a misinterpretation of laboratory results and, in turn, for the transition from the laboratory bench to the patient’s bed. For example, if it is known that the enzyme lactate dehydrogenase (LDH) is of exclusively cytosolic localization and the metabolite L-lactate (L-LACT) is a metabolic waste product—as the textbooks still declare—one would never expect oxidation of L-LACT in the mitochondria. It would be ruled out a priori, and yet that is what happens. Not only that, but L-LACT is also the most important gluconeogenetic precursor, not only in the liver. In the last century, it was demonstrated that L-LACT, produced by glycolysis during muscular effort, is transported to the liver, where it is used for the synthesis of new glucose by gluconeogenesis [119,120,121]. The transport of glucose from the liver to the muscle, where it is used for glycogen synthesis or consumed in glycolysis, completes the Cori cycle, named after its inventor [119]. If until recently it was believed that the accumulation of lactate in skeletal muscles was a consequence of anaerobic metabolism, which occurs when the energy needs of the tissues exceed their ability to oxidize the PYR produced in glycolysis, metabolic studies suggest that L-LACT is actually an intermediate and not a terminal product of waste. Indeed, these studies demonstrate that, even in fully oxygenated muscle tissue, L-LACT is continuously produced and used: it is actively oxidized by the mitochondrion of skeletal muscle, and, during physical exercise, oxidation can provide up to 75% of the removal of L-LACT, while the remainder is used in gluconeogenesis. In this regard, in 2009, Brooks, of the University of California at Berkeley, brilliantly deduced that lactate, continuously produced and removed, circulates between all cells in the body, suggesting the existence of the cell-to-cell lactate shuttle [122]. Thus, in addition to the Cori cycle, the lactate shuttle hypothesis proposes a complementary function of lactate in many tissues. It is produced and used by cells both in aerobic and anaerobic conditions for the maintenance of a dynamic balance of energy substrates [123]. What was long thought to be the athlete’s poison, which accumulates in the muscles and leads to muscle fatigue, reduces performance, and generates pain, has turned out to be a source of energy, coming to represent lactate, the link between oxidative and glycolytic, or anaerobic, metabolism. Next, Brooks, together with his collaborators Hashimoto and Hussien [124], showed in skeletal muscle cells the co-localization within the mitochondria of three critical elements of the lactate pathway: (*i*) the L-LACT transporter protein; (*ii*) the mitochondrial enzyme L-lactate dehydrogenase, which catalyzes the first step in the conversion of lactate into energy; (*iii*) mitochondrial cytochrome oxidase, the protein complex in which oxygen is used, proposed a lactate oxidation process within the mitochondria [124]. Subsequently, the role of mitochondria in L-LACT metabolism has been demonstrated in sperm, liver, heart, skeletal muscle, brain, plants, and yeast [125].

Here we just limit ourselves to saying that there is the presence of the enzyme L-lactate dehydrogenase inside mitochondria, where L-LACT enters through carrier-mediated transport processes. Functional studies have demonstrated the presence of different carriers for L-LACT (see [125]), suggesting that the mitochondria play an important role in the metabolism of L-LACT, which has also been investigated in particular pathological conditions. In tumor cells, there is an increased production of this metabolite, as has been known for decades. Using human hepatocarcinoma cells (Hep G2) as a model system, the presence of L-LACT in the mitochondria was demonstrated by immunoblotting, confocal microscopy, and an enzymatic activity assay. Using spectroscopic techniques, it was demonstrated that L-LACT can not only exit, as reported in the literature [126,127], but also enter these cells and, together with the L-LACT formed in the cytosol, access the mitochondrion to be oxidized to PYR. These discoveries suggest an anaplerotic role for L-LACT in tumor cells and provide new elements of knowledge in the metabolism of these cells, which cannot be disregarded in the development of new strategies for the fight against this pathology. Furthermore, interesting is the presence in the IMS of a lactate oxidase [100], which could be a candidate for the new role proposed for L-LACT as “lactormone”, i.e., in Brooks’ term [128], as a cell-signaling molecule that is involved in the adaptive response to exercise.

## 3. The Mitochondria Get Sick

The first major wave of mitochondrial research, as briefly described above, extends from the 1940s, with the pioneering work of Lehninger, Chance, Slater, Ernster, and others, through the “chemiosmotic revolution” initiated by Peter Mitchell, to the early 1980s. During this period, basic mitochondrial functions were identified and characterized with increasing precision, and the organelle emerged as a beautifully self-regulating machine for ATP generation (see [13,129]).

Only in recent years has a large body of scientists, both biological and biomedical, come to recognize that mitochondria are actually involved in many other important processes. One of these is cell death, which, not surprisingly, is a process that partially involves the OXPHOS machinery.

The mitochondrion, as an energy control hub, represents an important driving force decisive for many physiological and pathological situations, so much so that the biology of the mitochondria continues to assume an increasingly relevant role in medicine, as well as growing scientific evidence demonstrating how the mitochondria play critical roles in the pathogenesis of many diseases. 

After all, if we consider that, beyond their “energetic” role in ATP synthesis, the mitochondria are at the crossroads of multiple metabolic pathways that take place in the mitochondria and redirect carbohydrates, amino acids, and lipids in the cellular pathways intended for the biosynthesis of macromolecules, the de-novo synthesis of lipids or nucleotides, and the cellular antioxidant defenses [13]. It follows that the ‘block’—intended as malfunctioning or even the interruption of functioning—of specific enzymes and/or mitochondrial translocators causes the accumulation of metabolic intermediates, which, in turn, can cause deleterious effects on cellular and physiological functioning, laying the basis for the manifestation and development of pathological symptoms.

The experimental procedures of mitochondrial isolation from major tissues (heart, liver, and brain) have provided the cornerstone of our knowledge base on mitochondria and their functional characterization. However, if we are to understand the way in which mitochondria behave in relation to other aspects of cellular physiology and how other cellular functions respond to changes in mitochondrial function, then we need experimental models in which these processes can be studied in intact cells. In some studies that have investigated the role of the mitochondria in disease, the cellular homogenate has been used as an experimental model precisely because cellular metabolism is closely interconnected with mitochondrial functionality, which cannot be ignored. It follows that the measurements, previously carried out using isolated mitochondria, have been miniaturized. A clear example of two pathways intertwined through thermodynamic and kinetic constraints and strictly involved in cellular energy metabolism is represented by glycolysis and OXPHOS [130]. Glycolysis in the cytosol and OXPHOS in the mitochondria, linked by the pyruvate dehydrogenase complex, are the two main metabolic pathways that produce energy (Figure 1). However, for the cell to function properly, the two metabolic pathways must be able to dialogue and use the products of one pathway as intermediates for another. An understanding of the main metabolic pathways, in light of the existence of cross-talk between cytoplasm and mitochondria, could lead to a harmonic understanding of cellular biochemistry and stimulate further interest in studying metabolism in intact cells/organelles. In this regard, no textbook ‘dares’ to go a step further, that is, to expose itself in the description of cellular metabolism, describing the cooperation of enzymes and translocators.

All the topics covered in textbooks for future biologists and physicians are presented in an aseptic way, detached from the context in which they operate, which, in fact, is the only one that gives meaning to existence as well as to the role that the mitochondria assume in the cell. A reductive view is adopted not only of the role of translocators but of the entire mitochondrial environment within the cell.

The existence of a link between mitochondria and some diseases began to become evident in 1975, when Douglas Wallace and his colleagues at Yale University described an association between mitochondrial DNA and a genetic disease [131]. In the 1990s, the effects of mitochondrial DNA mutations were linked to several other diseases (see [132,133]). One in 5000 people has an inherited mitochondrial disease of some kind, with consequences ranging from diabetes to vision and hearing problems to learning disabilities and other dysfunctions. However, it is only in the last decade or so that science has begun to carefully study the influence of mitochondria on health and well-being, with a particular focus on stress, anxiety, and depression.

### Involvement of the Mitochondria in Diseases, Rare and Not

Biochemical modifications of tau proteins—together with highly toxic Amyloid βeta (Aβ)-1-42 aggregated—have been designated to be among the earliest neurobiological changes and histopathological features of AD. In 2008, in a study aimed at decoding the functional role of the N-terminal domain of tau, Amadoro et al. found that high intracellular levels of N-terminal tau fragments lacking the first 25 amino acids, such as the NH2-26-44 tau fragment, evoke a potent neurotoxic effect in primary neurons of the hippocampus and cortex [134], inducing a necrotic type of cell death, sustained by sustained activation of the extra-synaptic N-methyl-d-aspartate (NMDA) receptor. The NH2-26–44 tau fragment-dependent mechanism that underlies the necrosis was to be deciphered. Thus, investigating OXPHOS that was dramatically impaired by this NH2tau fragment, with the ANT as the sole mitochondrial target responsible for OXPHOS impairment, Atlante et al. provided an explanation for the reduced ATP availability in the cytosol—proposed as a cause of stimulation—mediated neuronal death by excessive and prolonged use of NMDARs in AD [135]. Indeed, abnormal activation of NMDAR is believed to result in the release of glutamate from the cell due to Na^+^-coupled electrogenic transporter reversals and hence excitotoxicity (see [135]). This is a classic ‘fortuitous’ example for which, by doing research—in this specific case, investigating the toxicity of the tau fragment—we came across the mitochondrion, further validating what was a hypothesis at the time—but now it is a certainty—that among the most serious and important neuropathological alterations of AD, mitochondrial dysfunction is among the timely events in the onset of the disease itself as well as in its progression.

Far from intending to argue the issue of mitochondrial dysfunction in AD, to which many studies by illustrious neuroscientists are dedicated [136,137,138,139,140]. We also want to submit to the reader’s attention a singular study that has allowed us to experimentally demonstrate, beyond the impairment of mitochondrial metabolism, which had already been well documented (see [141]), the mitochondrial quiescence: the mitochondria are sent to quiescence in a cell in which the disease is taking root, in the unsuccessful attempt of the cell itself to oppose the imminent death. We came to talk about mitochondrial quiescence by observing that cells in which apoptosis was induced—a condition that mimics AD in two different phases, namely the ‘early’, corresponding to the onset of the disease, and the ‘late’, corresponding to the advanced phase of the disease—were insensitive to the inhibitory effect of compounds that block mitochondrial RC [99]. Differently, the same compounds, at the same concentration, determine the death of control cells. If, on the one hand, the mitochondria were insensitive to the inhibitory action of the tested compounds, on the other hand, we witnessed a reprogramming of glucose metabolism by the cell, corresponding to the activation of the Warburg effect, a typical metabolic condition of tumor cells [99,142,143]. Thus, in the initial stage of apoptosis, glucose metabolism is enhanced, i.e., the key proteins that internalize and metabolize glucose—glucose transporter, hexokinase, and phosphofructokinase—are upregulated, concomitant with a parallel decrease in oxygen consumption by mitochondria—they fall asleep—and increased accumulation of L-LACT. The loss of the adaptive advantage offered by aerobic glycolysis, which occurs in the late phase of apoptosis, exacerbates the pathological processes underlying neurodegeneration, inevitably leading to cell death. Therefore, both aerobic glycolysis, i.e., the Warburg effect, essentially due to the protective numbness of the mitochondria, and anaerobic glycolysis, caused by mitochondrial impairment, characterize the entire time frame of apoptosis, from the initial to the late phase, which mimics the development of AD [99,141]. This scenario suggests that the development of two diseases that—it seems—are initiated by an alteration of apoptosis—AD and cancer—proceeds through a common molecular mechanism: a metabolic shift of glucose utilization from OXPHOS to glycolysis, with production of lactate even in the presence of oxygen, thus realizing a ‘‘sweet tooth’’ phenotype.

Before pointing out the difference between cancer and AD, the significance of mitochondrial dysfunction in cancer deserves a mention, since dysregulated mitochondrial function was recently added as one of the cancer hallmarks and, in addition, it has been proven to be essential for tumorigenesis, tumor development, and tumor metastasis [144,145].

Mitochondria are important mediators of tumorigenesis. Many facets of mitochondrial biology beyond energy production actively contribute to the acquisition of typical malignant traits, including mitochondrial biogenesis and dynamics, cell death susceptibility, oxidative stress, and metabolic regulation [146]. Deregulation of mitochondrial metabolism can generate oncoproteins and pro-tumor metabolites due to dysfunctional anabolic and catabolic processes. In particular, selective mutations identified in the mitochondrial enzymes considered tumorigenic in many types of cancer are mainly part of the OXPHOS apparatus or TCA cycle but are also involved in other biosynthetic pathways (for refs, see [147]). This significantly contributes to the development of multiple types of human cancer (for refs, see [148]).

A century ago, precisely in the 1920s, Otto Warburg observed that cancer cells rely on glycolysis even under normoxic conditions. Although at the time Warburg hypothesized that the switch to aerobic glycolysis resulted from dysfunctional mitochondria, pointing to this metabolic change as a causative event leading to cancer, it was later demonstrated that functional mitochondria are essential in many types of cancer and even that deleting the mitochondrial DNA of cancer cells reduces the growth rates and carcinogenicity of those cells [149,150,151]. It is now believed that, since cancer cells are highly proliferative, their metabolic needs—i.e., the availability of metabolites such as nucleotides, amino acids, and lipids serving as biomass precursors (see [152])—are different from those of differentiated cells, which rely on OXPHOS for energy production. Mitochondrial dysfunction could then trigger the change in energy metabolism of cancer cells from OXPHOS to glycolysis, thus contributing to cancer progression. However, a significant portion of their ATP is still produced via OXPHOS, suggesting that at least some mitochondrial function is preserved in cancer [67,152,153].

Back to the point of questioning the difference between cancer and AD: (1) in cancer, the increased availability of glucose inside the cell stimulates glycolysis and, in turn, the mitochondrial and cytosolic pathways associated with it in order to “use” the excess cellular glucose in the most correct way. This means that the upregulation of glucose uptake and thus glycolytic flux is a primary alteration in cancer and not a consequence of altered mitochondrial function; (2) in AD, it is evident that glycolytic flux and mitochondrial function are closely intertwined, but neurons respond to the perturbed cellular conditions by primarily enhancing aerobic glycolysis and causing a “freeze” of the mitochondria; the switch from OXPHOS to glycolysis is understood only as an attempt to save neurons from death, an attempt that proves to be transient because it ultimately fails, probably due to irreversible mitochondrial damage that occurs later in the course of the disease. After all, if we pause for a moment on the metabolic peculiarity of neurons, they can neither function properly nor survive without OXPHOS, since neuronal activity requires a high level of energy and therefore strictly depends on the oxidation of glucose by the mitochondria.

Actually—this aspect is known to everyone, scientists and non-scientists alike—AD is still without a cure, i.e., there are no ‘disease-modifying’ therapies. With a bioinformatics and experimental approach, a study published in Nature [154] demonstrated that enhancing mitochondrial function and proteostasis can reduce the formation of harmful protein aggregates in the context of proteotoxic disease, characterized by reduced mitochondrial activity and loss of proteostasis, supporting the concept that enhancing mitochondrial proteostasis may hold promise for managing pervasive Aβ proteopathies, such as AD. This is why research on mitochondria as a metabolic contributor to AD has soared.

Moreover, in a fairly recent study, Stanga et al. dissected the pathways related to mitochondrial dysfunctions that are shared between several neurodegenerative diseases, such as Alzheimer’s, Parkinson’s, Huntington’s, amyotrophic lateral sclerosis, and spinal muscular atrophy. The authors observed how the same dysfunctions unite the different pathologies, confirming the hypothesis that the mitochondria represent the ‘fil rouge’ that unites them and identifying the mitochondrial function as the “switch” responsible for the passage from a normal physiological condition to a degenerative one [155]. Mitochondria, therefore, can represent an excellent target for the development of innovative and transversal therapeutic strategies capable of acting on multiple pathologies by exploiting common mechanisms.

In this regard, it seems that the damage to the mitochondria of the brain cells of individuals with Down syndrome (DS) favors the development of AD [156]. The DS phenotype is highly complex with constant features, such as mental retardation, dysmorphic features, and hypotonia, and variable features, including heart defects, susceptibility to AD, type 2 diabetes, obesity, and immune disorders. To date, the hypothesis most supported by the data is that oxidative stress caused by altered cellular metabolism is one of the main causes of neurological damage in DS [157,158,159]. Starting from this observation, scientists are working in an attempt to develop a prenatal treatment aimed at reducing OS and neuroinflammation to thus improve the brain development of people with DS and avoid the possibility of developing AD—already at a young age.

In the decades following Lejeune’s discovery in 1959 [160] of the genetic cause of DS, i.e., the trisomy of chromosome 21, Lejeune himself hypothesized as responsible for the syndrome the excess production of molecules toxic for the cell; however, research concentrated on the role of the extra chromosome 21. The initial Lejeune’s hypothesis, impossible to verify at the time, subsequently proved to be founded and critically responsible for a state of metabolic and functional alteration of the neuronal cells leading to intellectual disability. Therefore, the goal was precisely to identify the causal link between genetic and functional alterations in DS. It is easier to act on the impaired metabolism than on the genes. However, one of the most important problems in studying mitochondrial dysfunction in disease is dissecting the multistep processes leading to cellular energy deficits in order to identify the component/molecule responsible for the dysfunction. To understand the relationships between different cellular components in a complex biological system, it may be more appropriate to consider functional products rather than genes, in light of their specific expression under different conditions (e.g., tissue, developmental stage, or pathological state) [161]. Studies based on the proteomic approach (see [162,163]) also appear to be limited by the impossibility of assessing both the activity of the altered proteins and the rate-limiting step of that specific metabolic pathway. To date, those few active DS research groups are increasingly decoding the secrets of that extra chromosome on which that altered cellular metabolism depends.

Some mitochondrial functions such as respiratory capacity, membrane potential generation, and ATP synthesis have been found severely compromised in human skin fibroblasts with chromosome 21 trisomy due to an impairment in OXPHOS machinery involving mitochondrial complex I, ATP synthase, ANT, and adenylate kinase activities, attributed to cAMP/PKA-mediated post-translational impairment [164,165]. Regarding ROS, their overproduction by dysfunctional mitochondria in DS human skin fibroblasts correlates with a drastic selective reduction in the catalytic efficiency of RC complex I, confirming a mitochondrial origin of OS in DS [166,167]. Upregulation of ROS generation and mitochondrial dysfunction have been identified in DS cells as early as embryonic life [168]. In fact, OS markers are already present in the amniotic fluid of trisomy fetuses during pregnancy [169]. Interestingly, these mitochondrial defects are present in all DS cell types analyzed so far, from peripheral to CNS cells (see [167]), suggesting that mitochondrial dysfunction is an intrinsic feature of the syndrome (see [167]). Added to this, according to the recent observation by Bordi and coauthors, is the fact that primary human fibroblasts derived from individuals with DS are deficient in mitophagy [170], thus leading to the accumulation of damaged mitochondria with a consequent increase in OS. Since mitochondrial dysfunction and OS play a central role in the pathogenesis of AD [171], the extent of mitochondrial impairment is likely to be a risk factor for the early development of this deleterious neurodegenerative disease within the DS population.

Although more knowledge is needed to understand the role of mitochondrial dysfunctions in the composite cellular and molecular events leading to the intellectual deficit and neurodegeneration that occur in DS, combined therapeutic strategies that promote mitochondrial bioenergetics and prevent OS may be beneficial for the management of some of the clinical manifestations commonly associated with DS. Mitochondria-targeting molecules and nutrients, such as coenzyme Q (10), acetyl-l-carnitine, α-lipoic acid, tocopherol, and ascorbic acid, which support mitochondrial functions and reduce OS, have already been extensively studied for treatment of DS (reviewed in [172,173]). Furthermore, the ability of epigallocatechin-3-gallate—a member of a family of natural polyphenols, present in large quantities in green tea leaves—in cultured lymphoblasts and fibroblasts from DS subjects was tested: mitochondrial complex I and ATP synthase catalytic activity were rescued, OXPHOS efficiency was restored, and OS was contrasted [174]. Resveratrol also reverses the severe impairment of mitochondrial bioenergetics and biogenesis in hippocampal progenitor cells in the Ts65Dn mouse model of DS [175].

Another genetic disorder surprisingly involving the mitochondria is cystic fibrosis (CF), characterized by severe pulmonary dysfunction caused by mutations in the gene coding for the cystic fibrosis transmembrane conductance regulator (CFTR) and responsible for abnormal mucus secretions [176]. The normal CFTR gene acts through the production of a normal CFTR protein, localized on the apical membrane of the epithelial cells that line the ducts and cavities of many organs of our body, constituting a kind of channel that favors the passage of chloride (but also of other electrolytes) from inside these cells to the outside, resulting in the secretion of water. The defective CFTR gene produces a defective CFTR protein. Today, 2000 mutations of the CFTR gene are known. The most frequent in all populations is the ∆F508 (or F508del) mutation. Depending on the type of mutation, there are different effects on the CFTR protein: some mutations cause it not to be produced at all, while others allow a poorly functioning or reduced quantity of protein to be produced. Unfortunately, the ultimate effect on the CFTR protein and therefore on its clinical consequences is not known for all mutations. Until a few years ago, the paucity of information on the role of mitochondria in this insidious disease was mainly attributed to the fact that the primordial suspicion that the mutated protein responsible for the disease was mitochondrial had been dispelled. Researchers did not take into account the hypothetical involvement of the mitochondria, essentially focusing the main research on the protein encoded by the mutated CFTR gene, and, erroneously or due to extreme superficiality, the right weight was not given to the hypothetical involvement of the mitochondria.

In order to assess the involvement of mitochondria in CF, Atlante et al. have investigated some steps of the OXPHOS process by ascertaining what, if any, the relationship is between the activity of the mitochondria and the F508del mutant CFTR protein, with the ultimate goal of identifying possible new therapeutic targets aimed at recovering this protein. ANT-dependent ADP/ATP exchange, oxygen consumption, generation of mitochondrial membrane potential, and both mitochondrial Complex I (mtCx-I) and Complex IV activities were found to be impaired in CF cells associated with ROS overproduction and increased mitochondrial membrane lipid peroxidation (for refs, see [31]). In this context, it was also seen that mtCx-I-dependent and rotenone-sensitive ROS production is increased in CF, thus suggesting that the mitochondrial defect attributed to mtCx-I results from both a loss of activity and an increase in ROS production [177]. Regarding this topic, Kelly Aubert had previously observed that the decrease in mtCx-I activity in CF cells and CFTR knockout mice was restored to control values by treating the cells with GSH monoethyl ester, a membrane-permeable GSH analog capable of increasing mitochondrial GSH in different cellular models [178]. Furthermore, consistent with this, the existence of mitochondrial OS in the lungs of CFTR knockout mice was confirmed by both an increase in mitochondrial DNA oxidation and a loss of aconitase activity [179]. In addition to the mtCx-I defect, Atlante et al. also observed a functional defect in complex IV, strongly dependent on the membrane lipid environment (see above) [177]. Thus, since mitochondrial ROS production and membrane lipid peroxidation are increased in CF cells, Atlante et al. suggested that ROS-mediated damage of the membrane microenvironment might be responsible for complex IV inhibition. That treatment with CFTR correctors, i.e., VX-809 and TMA, compounds that increase the amount of functional CFTR on the cell surface, partially restores mitochondrial function in CF cells strongly suggests that there are interaction dynamics between CFTR, mitochondrial (patho)physiology, and ROS [31].

A small digression in this regard: it has been since 1994 that A.A. studies mitochondrial bioenergetics in AD, and since 2008, D.V. studies mitochondrial energy dysfunction in the DS, but the understanding of the role and involvement of mitochondria in the disease has only been taking shape for a few years, highlighting new potential targets for the development of new therapeutic approaches against neurodegeneration or intellectual disability.

A recently published work has allowed us to lay the first foundations for under-standing how the regulation mechanism works in CF. This is a complex and articulated biochemical analysis, which is important since in CF it has long been known that there is an increase in the concentration of extracellular glucose in the surface fluids of the airways. This anomalous concentration is the concomitant effect of various factors studied in the work, and among them are those related to the metabolic processes of the cell [180]. The issue is important because the increase in extracellular glucose would favor the explosion of pulmonary infection from airway pathogens. Researchers sought to understand which of the implicated and competing processes, and which of the biochemical intermediaries of these processes, were involved in the increased glucose level in the surface fluids of the CF airways. The aim of the researchers was to answer the question: by modulating the activity of enzymatic proteins and/or the level of metabolites involved in the utilization of intracellular glucose, can a lowering of the extracellular glucose level in CF be obtained? The work demonstrates, through biochemical measurements, protein assays, and statistical analyses, that some of the key proteins in glucose metabolism are over-activated in conjunction with a decrease in oxygen consumption by the mitochondria [180]. Once again, the mitochondrion modulates the metabolism in a genetic disease that, at first glance, seems to have nothing to do with the mitochondrion. It was evident that the activity of the mitochondria plays a leading role in CF to the point that, paradoxically, by inhibiting their activity and therefore their request for glucose, it is possible to regulate the concentration of extracellular glucose in the right direction. Actually, we are dealing with a pure basic research study—albeit in the context of a disease—but it covers a topic that has been scarcely explored and of undoubted interest, with a view to creating conditions in the CF airways that are less favorable for the engraftment of pathogenic bacteria.

Behind the mitochondria, which are the masters of genetic and non-genetic diseases (see above), there is also the mystery of a galaxy of rare diseases—but not so much the genetic diseases of the mitochondria, which often have a devastating impact on the organism and on the lives of those affected. These mitochondrial diseases are a very heterogeneous group of diseases, both from the point of view of symptoms and from a genetic point of view, because there are more than a thousand potentially involved genes in both mitochondrial DNA and nuclear DNA, which produce proteins that are then transferred into the mitochondrion. The result is mitochondrial bioenergetic dysfunction, which leads to cellular energetic suffering up to the death of the cell itself. They can be primary if they are caused by mutations that directly affect the components of the RC or mitochondrial biogenesis. Secondary mitochondrial dysfunction can be caused by a mitochondrial impairment due to other metabolic functions, such as the Krebs cycle [181], fatty acid oxidation [182], or, for example, a dysregulation of intracellular calcium ion fluxes caused by an endoplasmic reticulum disorder (Wolfram syndrome) [183].

Unfortunately, there are no cures for most mitochondrial diseases. In some cases, such as coenzyme Q10 deficiency, the administration of particular substances may be useful; in others, special diets may be advised. Sometimes supportive therapies such as respiratory physiotherapy, speech therapy, and physical activity seem useful. Even if the “cure” is missing, however, what is not lacking is research with a single purpose: to provide cells with the mutated gene with a healthy copy of the gene, conveyed through a viral vector.

Since the organs most affected are the brain and skeletal muscles, the pathologies are often known as mitochondrial encephalomyopathies [184]. However, all tissues with a high energy demand, such as, in addition to the brain and muscle, the heart, endocrine pancreas, kidney, liver, and sensorineural epithelia, are particularly affected by these diseases, which can be present at birth but can also occur at any age. It could simply be the mitochondria that reveal our life expectancy to us. Replacing diseased mitochondria with analogous but metabolically functioning bacteria—we are referring in this case to synthetic mitochondria—could provide an unusual but effective therapy to cure these rare and, at the moment, largely untreated diseases. To do this, it is essential to first know which bacteria to use to mimic the re-evolution process in the laboratory. Knowing this would allow us to better understand some diseases that affect these organelles or even pave the way for the creation of synthetic mitochondria to be used in the medical and environmental fields. The preparation of artificial mitochondria represents just one of the great challenges of synthetic biology [185,186,187], one of the most fascinating and promising areas of frontier research.

We are facing a real revolution because that bacterium mentioned above, which has made itself useful in the economy of the house that hosts it, not only gets sick (see above) but even travels (see below), i.e., it leaves the cell to be engulfed by that cell that is malfunctioning. It is well known that in neurons, the mitochondria are transported from the cell body to the cell processes, or vice versa, towards the cell body, allowing the removal of damaged mitochondria or the reconstitution of healthy mitochondria [41,188]. Otherwise, the observation of the horizontal transfer of mitochondria between mammalian cells is more recent, thus challenging the current concepts of segregation and inheritance of mitochondria and mtDNA [41].

Transfer, in a broad sense, is a giant field in biology; just think of the transfer of information between nerve cells operated by neurotransmitters, whose message is recognized by the receiving cell and translated into biological responses in correspondence with a specialized structure called a ‘synapse’. After all, all nervous activities, from the simplest reflex activities to higher functions such as learning and memory, depend on the transfer of information between nerve cells and therefore on the number of synapses and their efficiency in releasing the neurotransmitter, a property that is finely tuned on the basis of the history of the neuronal cell and the intracellular environmental context. The same goes for the transfer of mitochondria. Another important aspect to consider in terms of analogy is that, similarly to mirror neurons, which allow us to physiologically explain our ability to behave in relation to others, so the mitochondria are as if they entered into empathy with their fellows.

Inspired by the symbiotic origin of mitochondria and the cell’s ability to transfer these organelles to damaged neighbors, many researchers have developed procedures to artificially transfer mitochondria from one cell to another [189]. Several studies have demonstrated that mitochondria can move between cells in vitro (for refs, see [190]). The transport of mitochondria from one cell to another has gained prominence in recent years as a significant mediator of cellular health and fitness. Several reports provide evidence that mitochondrial transfer occurs in vivo and is involved in different pathophysiological conditions such as tissue injury and cancer progression (for refs, see [191]). In this regard, it is hypothesized that cells probably possess mechanisms to trigger organelle exchange in response to injury signals from distressed recipient cells (Figure 1). However, the molecular signals that initiated this process remain unidentified [192]. Most studies used stem cells as mitochondrial donors, but some others also used immortalized cells or primary cells from the same or different species [193]. Surely, an intriguing question pertains to the degree of cellular damage required to initiate the intercellular transfer of functional mitochondria. Although the underlying mechanisms of the migration of mitochondria toward cells and tissues with damaged mitochondria are not fully understood, the transfer of healthy mitochondria ameliorates mitochondrial defects and, above all, aids in the recovery of cellular function [191,194,195,196,197]. Mitochondria transfer from mesenchymal stem cells not only structurally but also functionally repairs renal proximal tubular epithelial cells in diabetic nephropathy in vivo [198]. In addition, mesenchymal stem cell (MSC)-based treatment has been demonstrated to be beneficial for ocular degeneration. The intake of mitochondria through tunneling nanotubes resulted in an improved metabolic function in the recipient ocular cells, i.e., mitochondrial “donation” improved energy metabolism in recipient cells [199]. This data supports the consideration according to which MSCs have been valued as a promising cell source for regenerating tissues, especially corneal tissues and the retina [200,201]. Consistently, another study [202] observed that in mice subjected to transient focal cerebral ischemia, neurons released the defective mitochondria into the extracellular space, where they acted as a “help” signal and mediated neuron-astrocyte cross-talk after ischemic stroke. Astrocytes from ischemic mice are able to produce functional mitochondria and transfer them into neurons to promote cell survival and attenuate neurological deficits [203], thus indicating the existence of crosstalk between glial cells and neurons during ischemic stroke. Promoting intercellular mitochondrial transfer by accelerating neuronal release or astrocytic engulfment could be a potential and attractive therapeutic strategy for the treatment of ischemic stroke in the future.

Although most published work refers to mitochondrial transfer as a way to replace dysfunctional mitochondria, cells could also transfer dysfunctional mitochondria [204,205] that escape mitophagy. This may be particularly relevant in those diseases, such as AD or Parkinson’s, in which increased mitochondrial dysfunction correlates with progressive degenerative phenotypes [206,207].

One particular investigation revealed that AD mice treated intravenously with freshly isolated human mitochondria showed better cognitive performance than control mice; furthermore, a substantial reduction in neuronal loss and gliosis was also observed in mice treated with mitochondria [208]. This research on mitochondrial intercellular transfer between different cell types provides a potential therapeutic target to improve mitochondrial biogenesis in AD patients. It is conceivable that a revolutionary transplant technique could heal damaged organs, offering a glimmer of hope to those struggling to survive events such as cardiac arrests, strokes, and the like [194,209]. Supplementation of exogenous healthy mitochondria to replace damaged mitochondria improved cellular bioenergetics, reversed excessive ROS production, and restored mitochondrial function [210,211].

As far as cancer is concerned, a critical donor of healthy mitochondria to cancer cells are stromal cells. Mitochondrial transfer via tunneling nanotubes was found to increase chemoresistance to doxorubicin and survival [212]. In addition, mitochondrial transfer from bone marrow mesenchymal cells to acute myelogenous leukemia cells in vivo confers chemoresistance and survival [213].

Many in vitro and in vivo studies have also demonstrated that increasing mtDNA copy numbers effectively promotes microsatellite-stabilized colorectal cancer cell survival and metastasis [214]. In contrast, Bonekamp et al. [215] identified specific mitochondrial transcription inhibitors that can suppress tumor growth by disrupting mtDNA transcription. Therefore, increasing the number of healthy mitochondria in cancer cells does not necessarily mean improved cancer survival. In July 2018, The New York Times published an article entitled ‘Dying Organs Restored to Life in Novel Experiments. An unusual transplant may revive tissues thought to be hopelessly damaged, including the heart and brain’, tells the story of little Georgia, who, as a newborn, had a cardiac arrest that damaged a large part of her little heart. A condition that, unfortunately, did not offer the little girl much hope of survival, except thanks to a heart-lung machine that would have kept her alive for a very limited period of time. At Boston Children’s Hospital, the little girl underwent a mitochondrial transplant, an experimental therapy that consists of infusing these organelles into the damaged tissue. This new technique proved to be effective for the first time in reactivating heart cells following a cardiac arrest. The experimental therapy was aimed at infusing healthy mitochondria into damaged cells in the hope that the latter could reactivate and regain their normal functions. Basically, it consisted of isolating and harvesting a billion mitochondria from a tiny portion of healthy abdominal muscle and injecting them back into his damaged heart muscle [216,217,218]. Of course, a clarification is needed: to obtain any benefit from mitochondrial transplantation, the transplanted mitochondria must be viable and competent in terms of OXPHOS efficiency [219]. While it is true that mitochondrial transfer is aimed at saving recipient cellular functions, it must be viewed with caution, as it remains a moot point, i.e., whether the transferred mitochondria remain functional [220] (Figure 1). After all, if mitochondria originate from bacteria, mitochondrial DNA and their components are highly immunogenic; therefore, care should be taken in determining the nature and reactivity of the mitochondria used for mitochondrial transfer/transplantation because, depending on the nature of the mitochondria, intercellular and intracellular signals vary, determining the survival or death of recipient cells [192,221].

## 4. Conclusions

In the old days, the picture of the mitochondrial universe was much more simplified: the good and efficient mitochondria were making ATP, while the bad and deficient mitochondria were producing ROS. Mitochondrial ROS were, of course, the absolute evil; they increased disease, damaged genes, and made us age miserably. These good old days of simple biology are long gone in the wake of new discoveries, and we are increasingly on the crest of the wave. Nowadays, we can state that the mitochondrion has effectively stripped itself of the image of an organelle exclusively devoted to and only responsible for the production of energy to enlarge the horizon of its multifunctional tasks by playing important and crucial roles for our health and disease.

These legendary organelles, which arose about two billion years ago from the engulfment of a bacterium by an ancient eukaryotic cell [222], became responsible for the functions of respiration and energy production. Today, it can be affirmed “strictly and side by side” that the mitochondria know no boundaries. They also possess a myriad of additional multifaceted functions, including the buffering capacity of calcium (Ca^2+^) (for refs, see [192,221]), heat production [223], generation of ROS [224,225], modulation of growth, aging, and cell death [226,227], showing to be valuable tools for genealogical reconstructions [118], and much more. Last but not least, their involvement in the wide range of mitochondrial dysfunctions responsible for various diseases [228,229,230] as well as their mission to come to the aid of their fellows, even leaving the warmth of the house that hosted them to go to the rescue of their own defective fellows inside the receiving cell (Figure 1).

In the face of the avalanche of information regarding mitochondria that has engulfed us in recent decades—some of it has been considered in this study—we are optimistic about the potential of looking beyond current technological and conceptual horizons to better understand how bioenergetic and metabolic remodeling plays out in health and disease.

## Data Availability

Not applicable.
